# The retrogenesis of age-related decline in declarative and procedural memory

**DOI:** 10.3389/fpsyg.2023.1212614

**Published:** 2023-07-27

**Authors:** Chenwei Xie, Manson Cheuk-Man Fong, Matthew King-Hang Ma, Juliahna Wang, William Shiyuan Wang

**Affiliations:** ^1^Department of Chinese and Bilingual Studies, Research Centre for Language, Cognition, and Neuroscience, The Hong Kong Polytechnic University, Kowloon, Hong Kong SAR, China; ^2^Research Institute for Smart Ageing, The Hong Kong Polytechnic University, Kowloon, Hong Kong SAR, China

**Keywords:** memory, retrogenesis hypothesis, declarative memory, procedural memory, age-related decline in memory

## Abstract

The retrogenesis hypothesis proposes that the order of breakdown of cognitive abilities in older adults is the reverse of the developmental order of children. Declarative and procedural memory systems, however, have been empirically understudied regarding this issue. The current study aimed to investigate whether retrogenesis occurs in the developmental and decline order of the declarative and procedural memory systems. Besides, we further investigated whether retrogenesis occurs in declarative memory, which was tested through the recognition of familiar and unfamiliar items. Both questions were investigated by looking at 28 Chinese younger adults and 27 cognitively healthy Chinese older adults. The recognition memory task and the Serial Reaction Time Task were administered on two consecutive days in order to measure their declarative and procedural memory, respectively. The results showed older adults performed significantly worse than younger adults for both tasks on both days, suggesting a decline in both declarative and procedural memory. Moreover, older adults exhibited relatively preserved declarative memory compared to procedural memory. This does not follow the expectations of the retrogenesis hypothesis. However, older adults demonstrated superior performance and a steeper rate of forgetting for recognizing familiar items than unfamiliar items. This reverses the developmental order of different patterns in the declarative memory system. Overall, we conclude that retrogenesis occurs in the declarative memory system, while does not in the decline order of the two memory systems; this understanding can better help inform our broader understanding of memory aging.

## 1. Introduction

The order of breakdown of cognitive abilities in older adults is posited to reverse the developmental order of children. Jakobson (1941/1968), based on his observations on the phonological features of language users, proposed an enlightened hypothesis that “aphasic losses reproduce in inverse order the sequence of acquisition in child language” (p. 78). The reversion concept is not confined to the linguistic discipline. Before Jakobson, Ribot (1881/2012), a French psychologist who pioneered psychology as a science, proposed Ribot’s law of regression, i.e., “it is a well-known fact in organic life that structures last formed are the first to degenerate… in the biological world, dissolution acts in a contrary direction to evolution… the new perishes before the old, the complex before the simple” (p. 127). After Jakobson, Reisberg et al. (1999) developed the “mirror-image” idea between acquisition and aging, and coined the term “retrogenesis.” This development was based on the clinical, electrophysiological, neurophysiologic, neuroimaging, and neuropathologic evidence. Retrogenesis was defined as “the process by which degenerative mechanisms reverse the order of acquisition in normal development” (Reisberg et al., 2002, p. 202). For our purposes, we examine retrogenesis as the order of breakdown of cognitive abilities, specifically memory systems, in older adults such that it is the reverse of the developmental order of children.

There is a growing body of evidence that suggests retrogenesis is rooted in cognitive, neural, and genetic processes. One example which illustrated this was when children were asked to complete the Mini-Mental State Examination (MMSE), a screening test for dementia, the researchers found a positive correlation between MMSE score and mental age (Ouvrier et al., 1993). Similarly, when Alzheimer’s disease patients were asked to perform the traditional Piagetian measures, their MMSE scores were positively related to the Piaget levels (Matteson et al., 1996). Therefore, cognitive functioning appears to be acquired and lost in reverse order. Moreover, neurologic and neuropathological data suggested that on the micro-scale, cortical regressive changes of myelination often follow a reverse progression of maturation (Braak and Braak, 1996). Likewise, on the macro-scale, the distribution of cerebral degeneration in Alzheimer’s disease reversed the ontogenetic process (Brun and Gustafson, 1976). In addition, retrogenesis was discovered in gene expression in healthy samples. During fetal development, there is a wave of gene expression changes that are reversed in the early postnatal period. Most importantly, this pattern of reversals reoccurs half a century later in life in the form of neurodegeneration and aging (Colantuoni et al., 2011). Therefore, converging evidence confirms the retrogenesis hypothesis in both healthy and pathological population (Douaud et al., 2014). However, this is still conceptually and empirically under-identified in declarative and procedural memory systems in healthy older adults.

Declarative memory and procedural memory are two major components of the memory system (Cohen and Squire, 1980; Tulving, 1985; Ullman et al., 1997; Ullman, 2001c; Squire, 2004). Declarative memory refers to the rapid learning, representation, and use of arbitrarily-related information, which includes the active recall of previous experiences and conscious retrieval of factual knowledge about the world (Tulving, 1972; Ullman, 2001a, 2004; Squire, 2004; Kuhl and Damasio, 2013). Procedural memory supports the learning and implementation of perceptual-motor, and cognitive skills and habits, such as driving a car and playing skilled games (Ullman, 2001a, 2004; Ullman et al., 2020). Declarative memory primarily depends on the hippocampus and nearby regions in the temporal lobes (Ullman, 2001a, 2004; Lum et al., 2012). These structures not only support the encoding and consolidation of new information but also facilitate the retrieval of stored information. Procedural memory is subserved by the cerebellum, basal ganglia, and the associated circuitry (Ullman and Pierpont, 2005; Takács et al., 2018; Ullman et al., 2020).

During the early stages of life, declarative and procedural memory develop on different trajectories (Hedden and Gabrieli, 2004). The acquisition of declarative memory increases during childhood and plateaus during adolescence and early adulthood. In contrast, it appears that procedural memory is already robust in childhood and peaks around the age of adolescence (Lum et al., 2010; Finn et al., 2016; Ullman, 2020). There is, however, a lack of research regarding the trajectories of declarative and procedural memory in the late stages of life. While the development order of the memory functions is first procedural memory and then declarative memory, the order of deterioration is unknown. This makes it difficult to determine whether the deterioration order of these two memory functions reverses their developmental patterns.

Furthermore, different types of information might lead to different patterns of declarative memory, for a review, see Eichenbaum et al. (2007) and Diana et al. (2007). Previous research discovered that when asked if they had seen the objects before, children recognized more real objects than made-up objects after a delay of both 10 min and 24 h (Hedenius et al., 2013; Lukács et al., 2017). Real objects were familiar items that had already been acquired in the declarative memory system before participants performed the memory task. On the other hand, made-up objects were unfamiliar and last-in items in the declarative memory system that were introduced for the first time to participants during the task. Children were more resistant to forgetting real objects, compared with made-up objects. For older adults, we are only aware of one study which has investigated how these two types of objects were processed in older adults; the study reported that older adults failed to recognize or forgot more real objects after a delay of 5 min, compared to made-up objects (Reifegerste et al., 2020). These findings suggest that made-up objects are first-out items, as they are more likely to be forgotten by older adults. Therefore, within a short delay, the forgetting of different types of information in declarative memory systems follows the “last-in-first-out” principle, as predicted by the retrogenesis hypothesis. However, it remains unclear if older adults followed the “last-in-first-out” trajectory when recognizing different objects after a longer delay, especially since declarative memory is a type of long-term memory.

In the present study, we aimed to examine the effects of aging on declarative and procedural memory with both short and long delays. In line with previous research (Ullman, 2020), we hypothesized that older adults would exhibit degeneration in declarative and procedural memory after both delays. Moreover, we investigated whether the decline followed the retrogenic processes, i.e., whether older adults demonstrated a reverse pattern of the development of children on two memory systems. It was expected that the disintegration of declarative memory was earlier than that of procedural memory, as declarative memory matured in a later stage compared to procedural memory (Lum et al., 2010; Finn et al., 2016; Ullman, 2020). Last, we explored the role of object type in declarative memory during development and aging. Based on the retrogenesis hypothesis and previous studies in children (Hedenius et al., 2013; Lukács et al., 2017), we predicted that older adults would perform better at remembering items that had been acquired, such as real objects, than items that were unfamiliar, such as made-up objects, after both a short delay and a long delay.

## 2. Methods

### 2.1. Participants

28 Chinese younger adults (mean age = 24.1 ± 2.6, range = 19–30; 14 M) and 27 Chinese older adults (mean age = 68.0 ± 2.7, range = 65–76; 11 M) were recruited for the current study. The younger adults were students at The Hong Kong Polytechnic University, while older adults were recruited from the Shenzhen Associations of Senior Scientists and Technicians. The Montreal Cognitive Assessment - Beijing (MoCA-BJ) was used to evaluate the cognitive health of older adults. The cut-off score of MoCA-BJ was 22, at which point there was optimal sensitivity and specificity for the Chinese population (Yu et al., 2012). In the current study, the score of older adults was 26.85 ± 1.75, ranging from 22 to 29. Therefore, all of the older adult participants were cognitively healthy and were thus included in the analysis. All participants were Chinese native speakers and used Mandarin in daily life. The participants had no history of motor or neurological disorders and had a normal or corrected-to-normal vision. The study was approved by The Hong Kong Polytechnic University’s Human Subject Ethics Subcommittee (Reference Number: HSEARS20210303002). Prior informed written consent was obtained from each participant. An honorarium was paid to each participant.

### 2.2. Procedure

#### 2.2.1. Declarative memory

This study tested declarative memory using the Declearn Task, a recognition memory task developed by the Brain and Language Lab at Georgetown University, and modified specifically for Chinese participants. The task consisted of three phases. In the incidental encoding phase, participants were presented with pictures and asked to determine whether the presented object was real or made-up. In the initial recognition phase (10-min delay) and the delayed retention phase (24-h delay), participants were asked whether they recognize the objects from the previous encoding phase. Participants were not informed about the two phases and thus, would not intentionally memorize the objects from the initial phase. The accuracy rate was recorded and utilized in the data analysis.

**Figure 1:**
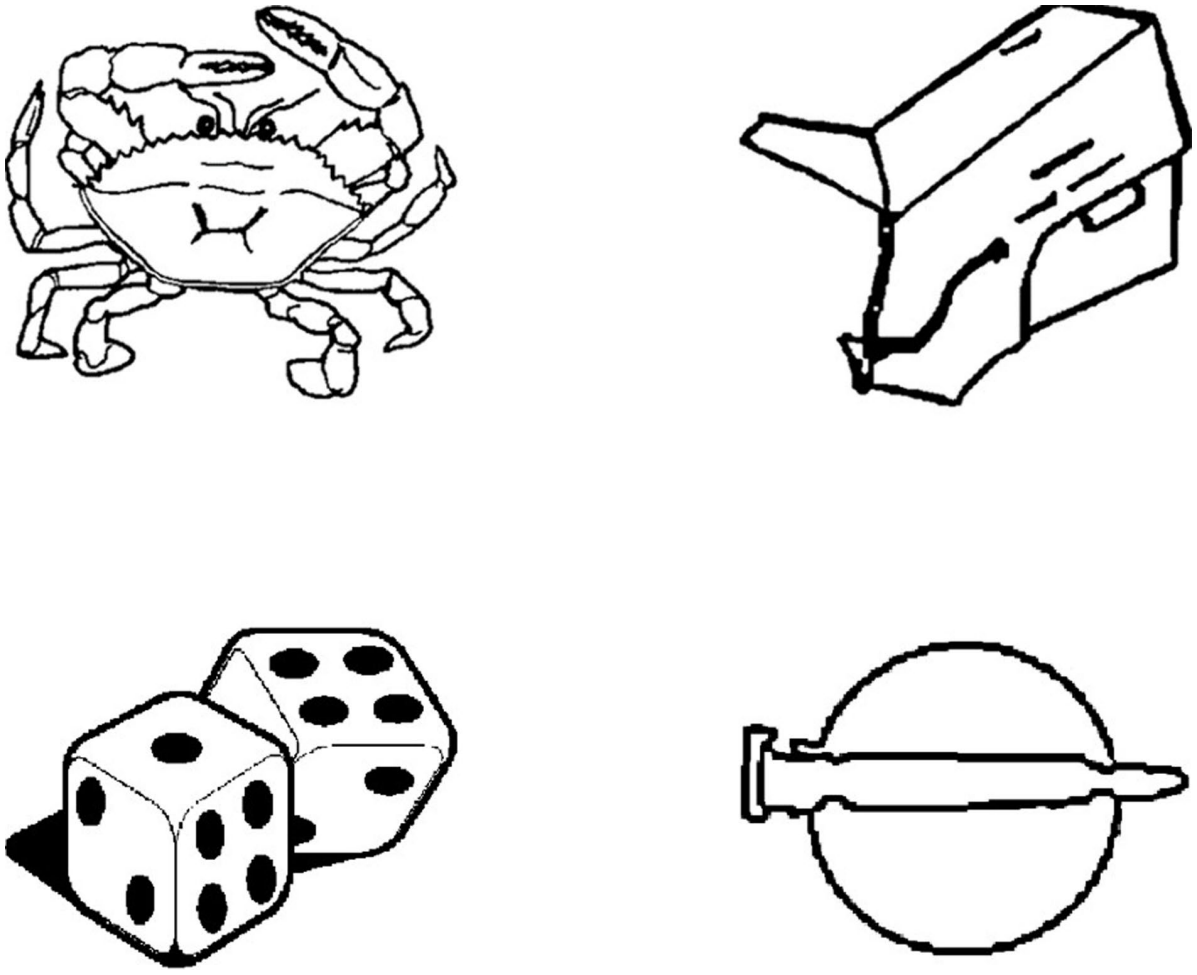
Examples of picture stimuli from the Declearn Tasks. The pictures on the left-hand side represent real objects. Those on the right-hand side represent made-up objects.

The picture stimuli were all black-and-white line drawings of made-up objects and real objects (Figure 1). Objects found in the real world were considered “real,” whereas objects created were considered “made-up.” For more detailed information on the picture stimuli (e.g., stimuli creation), see Hedenius et al. (2013). In each phase participants were shown 64 pictures, 32 real objects and 32 made-up objects. In the encoding phase, participants were shown an initial set of 64 pictures. In the recognition phase, half of the studied pictures and 32 new pictures were presented one at a time to participants. The new pictures served as foil stimuli. During the retention phase, the 32 pictures that were presented in encoding phase, but not shown in the recognition phase, were shown one at a time to participants. An additional 32 foil pictures were also introduced to participants during this phase. All picture stimuli were ordered in a pseudorandomized sequence, with no more than three consecutive real or made-up objects.

The pictures were presented on an LCD screen via a Surface Book 2 PC laptop running Windows 10, using E-Prime version 3.0. 1920 × 1,080 pixels were used in the display. Each picture was a 640 × 480-pixel image. The participants were seated approximately 100 centimeters away from the screen. Testing was administered in a quiet and light laboratory. Participants were instructed to make a response using their index and middle fingers on their dominant hand. To indicate their response, participants pressed the designated buttons on a multifunctional response and stimulus device (E-prime Chronos). The Chronos was placed on a desk in front of the participants.

Pictures were presented with the following presentation and timing parameters in all three phases, see Supplementary Figure S1 for the schematic illustration of the incidental encoding phase. At the beginning of each trial, a fixation cross appeared at the center of the display for 1,000 milliseconds (ms). The fixation cross was then replaced by the picture which stayed on the display for 500 ms. The picture was followed by a waiting interface which stayed up to 4,500 ms. Participants were instructed to respond as fast as possible during this period. As soon as participants pressed any buttons, the waiting interface was removed and followed by the fixation cross, signaling the next trial had started. A reminder then appeared on the lower left and lower right corners of the display in order to indicate the mapping of “real” and “made-up” to the Chronos buttons. During the recognition and retention phases, the reminder was changed to “yes” and “no.” Participants were given practice blocks to familiarize themselves with the task for all phases.

The recognition memory accuracy was assessed using d’ (d-prime), a method to reduce response bias. D-prime is equal to the z-transform of the hit rate minus that of the z-transform false alarm rate (*d*’ = z (hit rate) – z (false alarm rate)). The hit rate refers to the proportion of responses where the participant correctly answered “yes,” whereas the false alarm rate refers to the proportion of responses where the participant incorrectly answered “yes.” The d-prime was calculated *via* the psycho 2.2.3 package in R (Makowski, 2018) in which algorithms were formed based on Pallier (2002), and the adjustment for extreme values was made following the recommendations of Hautus (1995). Generally speaking, the higher the d-prime score, the better the ability of the participant to distinguish between old and new items.

The statistical analysis procedures of Hedenius et al. (2013) were followed to examine the declarative memory performance in the current study. First, a 2 (group: young vs. older adults) x 2 (day: day-one recognition phase after 10 min vs. day-two retention phase after 24 h) analysis of variance (ANOVA) with d-prime scores as the dependent variable was performed. This two-way ANOVA was to test whether there were significant differences in declarative memory performance between younger and older adults after short and long delays. Next, a 2 (object condition: real vs. made-up objects) x 2 (group: younger vs. older adults) ANOVA for the day-one recognition phase was performed. The ANOVA was aimed to examine whether the two groups differed in the effects of object condition after a short delay. The same ANOVA was computed for the day-two recognition phase to examine the role of aging in object condition effects after a long delay. Single sample *t*-tests were used to determine whether d-prime scores were significantly different than ‘0’, in which case the declarative memory of participants was used during the task. This mitigated the effect of chance in the analysis. However, if d’ was not significantly different than zero, then this would indicate the failing of the declarative memory.

#### 2.2.2. Procedural memory

The Serial Reaction Time Task (SRT) task was used to assess participants’ procedural memory capacity. The SRT task was executed by E-Prime version 3.0. During the task, participants were required to press the button of the E-prime Chronos that corresponded to the position where the visual stimulus (a yellow smiley face) would appear on the screen. The smiley face was displayed in one of four empty squares which were presented horizontally. Each square, from left to right, corresponded to buttons 1 through 4. Participants were instructed to place their index and middle fingers of both hands on the four buttons and respond only with these four fingers. Sequences were represented by the presentation order of the smiley faces and were divided into two types based on this order. The first type was sequential (S) which followed the predefined ten-item pattern: 1–3–4-2-3-1-4-2-1-4. The second type was random (R) for which there was no predefined pattern. Participants were not informed that they had been exposed to these two types of presentation orders.

There were two phases which were conducted on two consecutive days. Participants were not aware of a second phase until they had returned to the laboratory 24 h after the first phase. On the first day, participants attended the learning phase which was broken up into six blocks B1, B2, …, and B6, such that a given block designated a random (R) or sequential (S) sequence type. The sequence type for B1, B2, …, and B6 was ‘RSSSSR’ respectively. Each block contained 60 trials. The number of appearances of the smiley face in a given square, in the random blocks was equal to that in the sequential blocks. The following parameters were set according to Lum et al. (2012), the number of transitions between particular smiley face locations was equal in both types of blocks. For instance, since the transition ‘1–4’ occurred two times in the sequential block, it must also occur two times in the random block. The interstimulus interval (ISI) between trials was 100 ms. Furthermore, there was no time limit for button pressing, and the next trial was not presented unless participants provided a correct response or 20 incorrect responses. There were two practice blocks preceding the test block.

On the second day, the retention phase was conducted. The phase consisted of three blocks B7, B8, and B9 with the respective sequence types ‘RSR’. No practice block preceded the three test blocks. All parameters were maintained from the learning phase. Following the third block, participants were given a two-part recall task to assess their explicit knowledge of the sequence. During part one, Participants were first asked whether they had noticed a pattern within the blocks and to orally recite the pattern. Then they were given 20 s to press the buttons which corresponded to this same pattern. During part two, participants were asked to orally produce a sequence different from the pattern in part one. Finally, an additional 20 s were provided for participants to press the buttons corresponding to the new pattern. It should be noted that no participants noticed or correctly produced the predefined pattern.

Both the accuracy and reaction times (RTs) of participants were recorded. When participants were exposed to the predefined sequence repeatedly, they were able to learn it implicitly through their procedural memory. This task involved the procedural learning of perceptual-motor sequences. After the sequence was acquired, they were able to press the buttons in the pattern of the sequence at a faster rate. However, if the pattern was violated, participants would abruptly slow down.

This slowdown was recorded and compared in order to examine the differences in procedural memory learning between older and younger participants. In order to compare the RTs of the two groups, the RT scores were normalized using the *z*-score (by median) of each participant. This normalization controlled for variation in motor speed between the two groups, as aging is often associated with generally slower movements (Hoff et al., 2015). Only trials, such that the participant correctly responded, were included in this analysis. The same normalization method was previously used to control for variation between typically developing children and children with developmental language disorders, as well as younger and older adults when performing SRT tasks (Lum et al., 2012; Conti-Ramsden et al., 2015; Hoff et al., 2015). Attention to the task, due to its length and repetition, could also lead to misrepresentation in the analysis of some RT scores. In order to control for this, an RT was only included in the analysis if it was three or fewer standard deviations from the median. An average of 19.89 ± 16.51 data points (RTs) per older participant was removed from the analysis. An average of 20.36 ± 11.44 data points per younger participant were removed. There were 540 total data points per participant. There was no statistically significant difference between the two groups in terms of the removed data points [*t* (53) = −0.123, *p* = 0.903, Cohen’s *d* = −0.033].

The statistical analysis of procedural memory performance was based on the procedures of Lum et al. (2012). The accuracy of both younger and older adults was recorded, and it was determined that both groups were able to complete the task. Then a 2 (sequence type: sequential vs. random) x 2 (group: younger vs. older adults) ANOVA was computed each day with RT as the independent variable. These ANOVAs were to investigate whether, after short and long delays, the two groups differed in the rate at which they pressed each button.

Younger adults’ performance was used as a benchmark to test the retrogenesis hypothesis. In other words, compared to younger adults, if older adults performed better in one aspect but poorly in another aspect, it means the latter one was the first to show signs of decline. All calculation was performed using R 4.1.2 (R Core Team, 2021). The ANOVA was computed by the functionality of afex 1.1.1 (Henrik et al., 2022) and followed by post-hoc tests using package emmeans 1.7.1.1 (Lenth, 2021). A Greenhouse–Geisser adjustment for significance was applied to cases that failed to comply with the assumption of sphericity. The post-hoc pairwise comparisons were carried out if significant effects were found. *p* values were Tukey-adjusted for multiple *post hoc* comparisons.

## 3. Results

### 3.1. Declarative memory results

**Figure 2:**
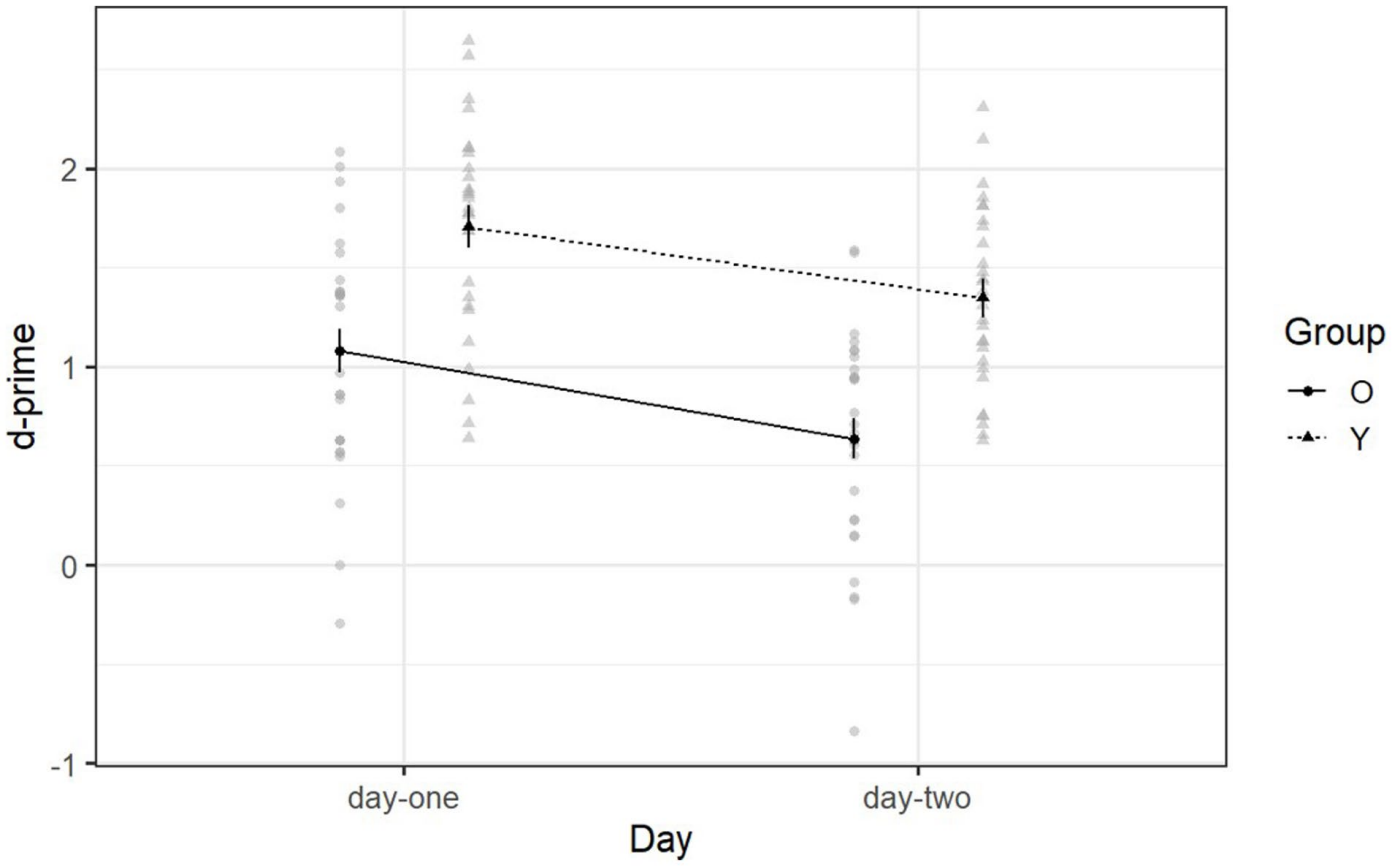
Mean d-prime scores of Declearn Tasks reported by group and day. Larger d-prime scores imply better declarative memory ability. If *d*’ = 0, then declarative memory has failed, i.e., participant’s performance is equal to chance.

Declarative memory ability is indicated by d-prime scores such that a larger d-prime score implies better declarative memory ability. Figure 2 shows the mean d-prime scores of each group for day one (recognition phase) and day two (retention phase). Younger adults (*M =* 1.53, SD = 0.95) performed better than older adults (*M =* 0.86, SD = 0.88) on day-one and day-two [*F* (1, 53) = 31.66, *p* < 0.001, *η*^2^_g_ = 0.28]. Both groups performed better on day-one (*M =* 1.40, SD = 1.04) than on day-two (*M =* 1.00, SD = 0.85) [*F* (1, 53) = 22.19, *p* < 0.001, η^2^_g_ = 0.12] (see Supplementary Table S1).

Next, we conducted 2 (object condition: real vs. made-up) x 2 (group: younger vs. older adults) ANOVAs for both recognition and retention phases to examine if there were differences in the effects of object conditions between the two groups.

**Figure 3:**
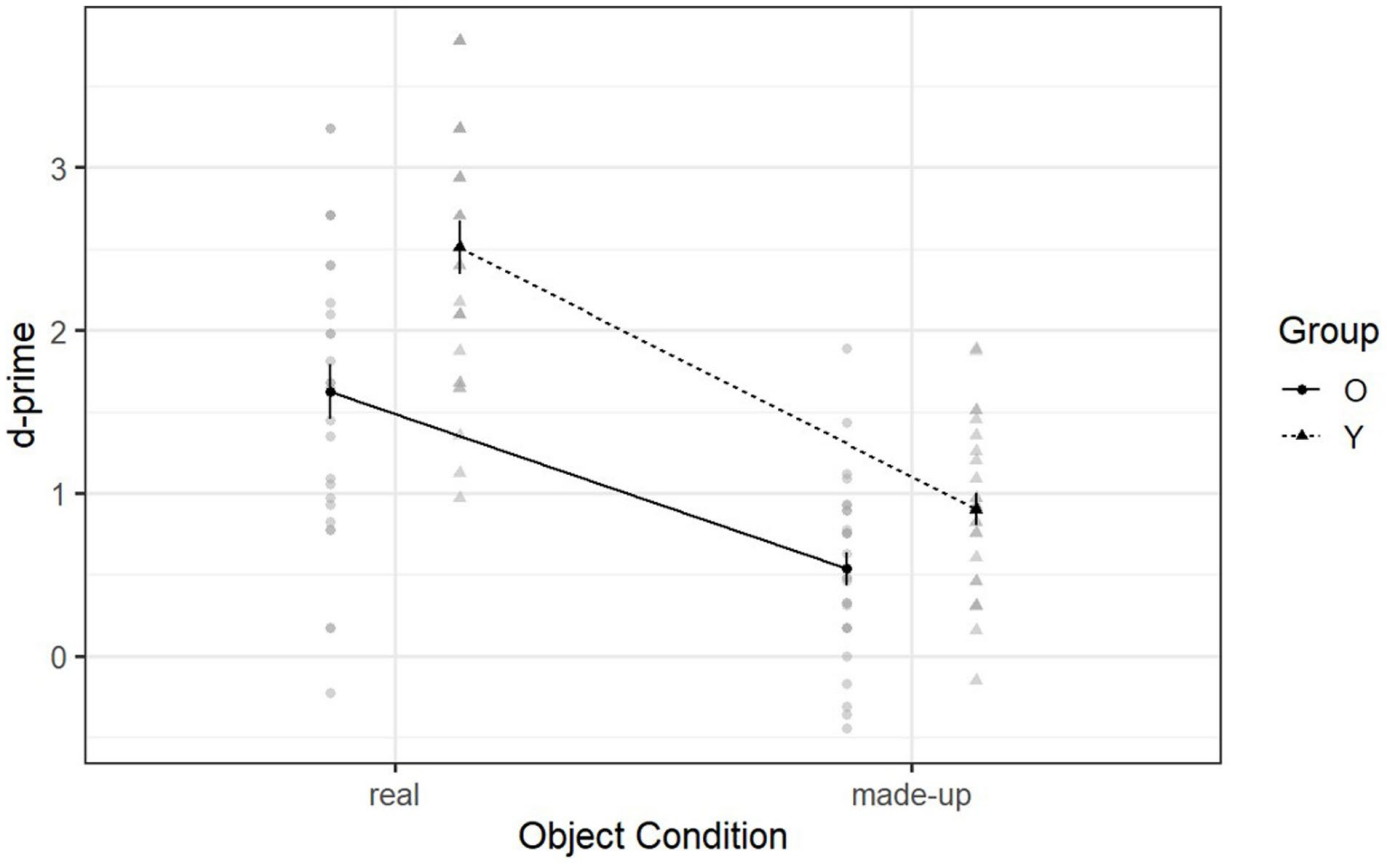
Mean d-prime scores of Declearn Tasks reported by group and condition on the day-one recognition phase.

Figure 3 illustrates the declarative memory ability of both groups in the day-one recognition phase. There was a significant interaction between group and object condition [F (1, 53) = 4.60, *p* = 0.037, η^2^_g_ = 0.03] (see Supplementary Table S2). Following up on this interaction, *post hoc* analyses revealed that younger adults (*M =* 2.51, SD = 0.84) and older adults (*M =* 1.62, SD = 0.92) had significantly different performances when recognizing real objects (*p* = 0.003). However, they had marginally similar performance when recognizing made-up objects (younger adults: *M =* 0.90, SD = 0.50; older adults: *M =* 0.54, SD = 0.55; *p* = 0.059) (see Supplementary Table S3). Thus older adults did not show a decline in declarative memory ability when recognizing made-up objects after a 10-min delay. Moreover, the single sample t-test indicated that older adults had significantly higher d-prime scores than the chance level (in which case *d*’ = 0) when learning made-up objects on the first day [t (53) = 8.51, *p* < 0.001]. This indicates that declarative memory was used by the older adults to learn the made-up objects after a 10-min delay.

**Figure 4:**
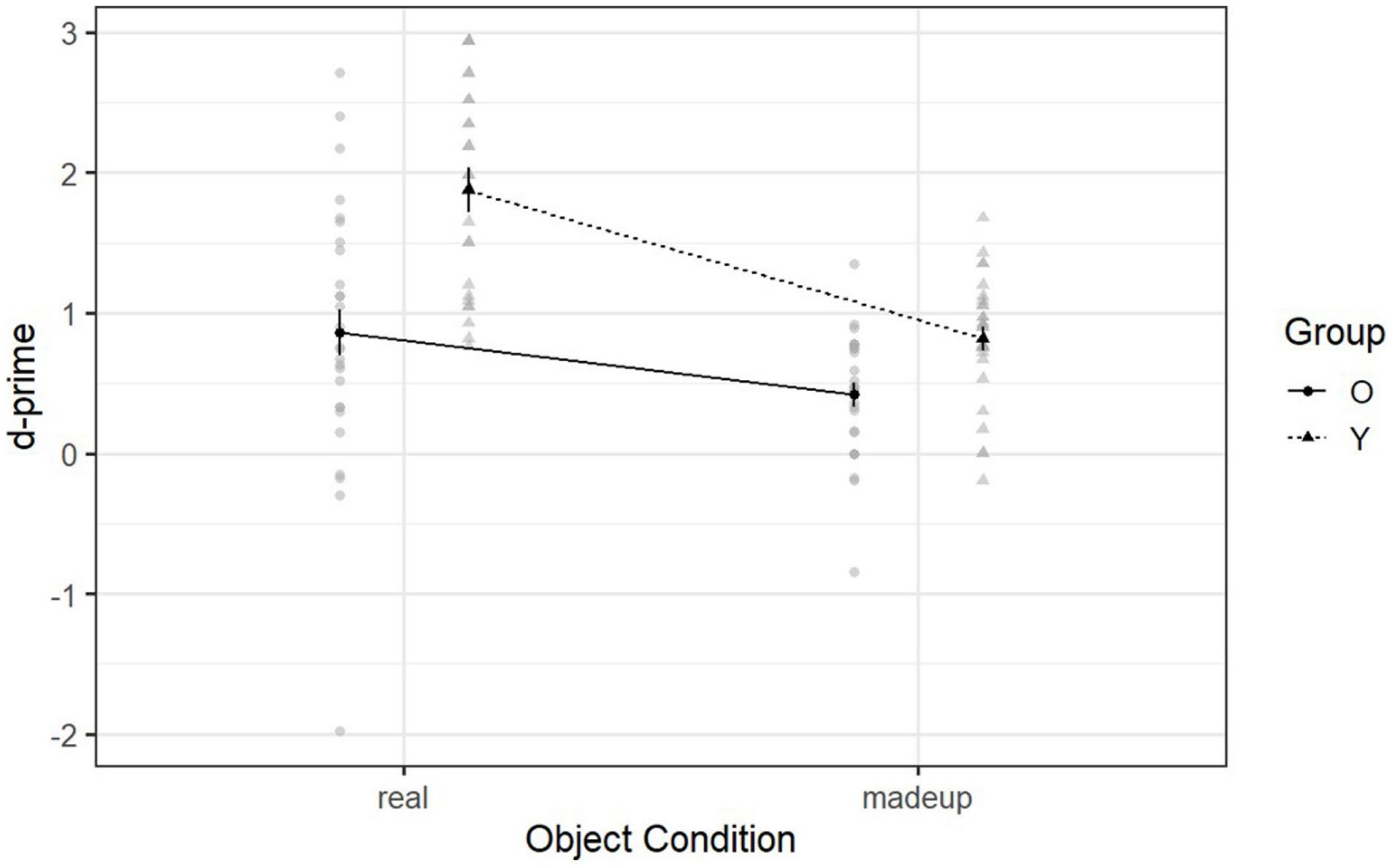
Mean d-prime scores of Declearn Tasks reported by group and condition on the day-two retention phase.

Figure 4 shows the declarative memory ability of the two groups in the day-two retention phase. Neither type of object was recognized by the younger adults (real objects: *M =* 1.88, SD = 0.69; made-up objects: *M =* 0.81, SD = 0.45) as well as it was by the older adults (real objects: *M =* 0.86, SD = 0.96; made-up objects: *M =* 0.42, SD = 0.44) [*F* (1, 95.88) = 5.69, *p* = 0.006, *η*^2^_g_ = 0.03] (see Supplementary Tables S4, S5 for details about the post-hoc comparisons). Thus the declarative memory ability, when recognizing made-up objects after a 24-h delay, was significantly different between the two groups. This contrasts with the day-one recognition phase, during which the two groups demonstrated similar declarative memory ability when recognizing made-up objects after a 10-min delay. Thus, declarative memory ability for made-up objects deteriorates after a long delay but not a short delay, whereas this is not observed in declarative memory ability for real objects even after a short delay. On the other hand, the single sample *t*-test indicated that older adults’ d-prime scores for made-up objects were still significantly above the chance level [*t* (53) = 6.06, *p* < 0.001], suggesting that they can still make use of declarative memory to support the retrieval of made-up objects after 24 h.

### 3.2. Procedural memory results

In this study, procedural memory was examined through the analysis of sequence learning in the SRT task. First, the accuracy of both groups was evaluated. Both younger adults (*M =* 96.8%, SD = 2.5%) and older adults (*M =* 97.4%, SD = 3.0%) performed with a high level of accuracy. Based on an independent samples *t*-test, no significant differences in accuracy were found between the groups [*t* (53) = 0.76, *p* = 0.45, Cohen’s *d* = 0.205], suggesting that older adults completed the SRT task as accurately as their younger counterparts.

**Figure 5:**
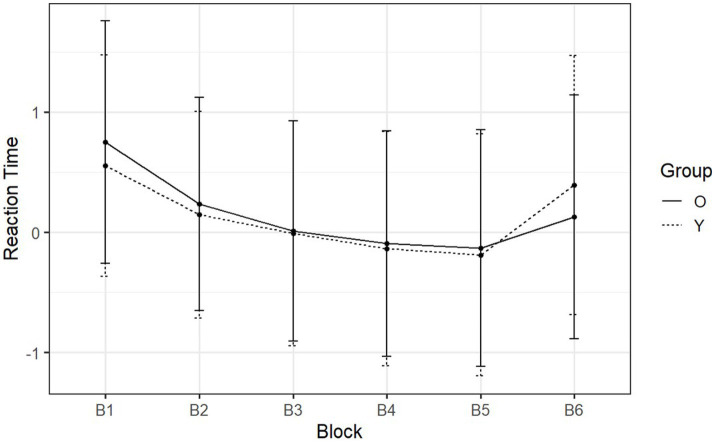
Day-one mean normalized RTs of SRT tasks reported by block and group. The *x*-axis is the six blocks with the respective sequence types ‘RSSSSR’. The *y*-axis is the reaction time (*z*-score).

**Figure 6:**
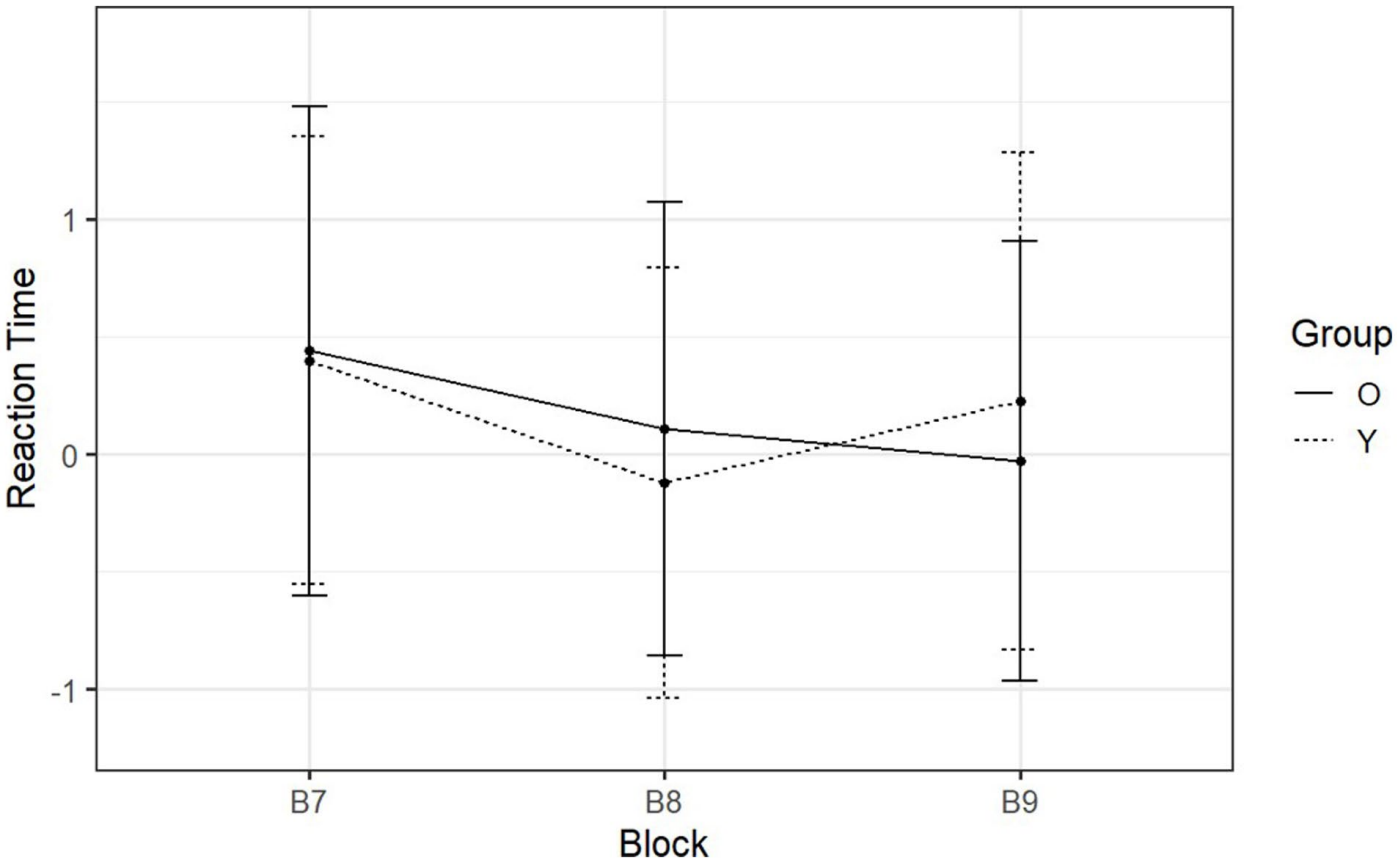
Day-two mean normalized RTs of SRT tasks reported by block and group. The *x*-axis is the three blocks with the respective sequence types ‘RSR’.

In the following section, RTs were examined, which were our principal dependent measure. The normalized RTs of day one (learning phase) and day two (retention phase) are shown in Figures 5 and 6 across each block. In the figure, the RTs are analyzed per group, and only correct responses are included in the analysis. Then we examined the change in RTs when participants transitioned from sequence type (S) to (R). This occurred in the transition from B5 to B6 (day-one) and in the transition from B8 to B9 (day-two). This was studied in both groups separately in order to investigate whether there was evidence of sequence learning for both or either group on each day.

On day one, participants transitioned from sequential to random between B5 and B6. What can be observed was a minimum RT at B5 (Figure 5) in both groups (younger adults: *M =* −0.23, SD = 1.02; older adults: *M =* −0.18, SD = 0.98). For B1 through B5, RT was strictly decreasing, i.e., the rate at which participants pressed each button was increasing. At B6 the sequence shifted from sequential to random, resulting in an increase in RT in both groups (younger adults: *M =* 0.15, SD = 1.08; older adults: *M =* 0.03, SD = 1.01). These significant increases demonstrated the acquisition of the predefined pattern during the sequential blocks (B2 through B5) [*F* (1, 53) = 59.27, *p* < 0.001, η^2^_g_ = 0.23] (see Supplementary Tables S6, S7 for details about the post-hoc comparisons). At this point, the RT increased by 0.58 (SD = 0.33) for younger adults and 0.30 (SD = 0.30) for older adults. The Welch two-sample t-test showed that the difference between how much each group increased was statistically significant [*t* (52.9) = −3.38, *p* = 0.001, Cohen’s *d* = −0.91]. Therefore, although older adults could still make use of procedural memory on day one, it was less effective than the procedural memory of younger adults.

On day two, participants transitioned from sequential to random between B8 and B9 (Figure 6). The two-way ANOVA yielded a significant interaction between group and sequence type [*F* (1, 53) = 35.31, *p* < 0.001, *η*^2^_g_ = 0.28] (see Supplementary Table S8). Following up on this interaction, *post hoc* analyses revealed that RTs of younger adults significantly increased during the transition from B8 (*M =* −0.12, SD = 0.92) to B9 (*M =* 0.23, SD = 1.06) (*p* < 0.001). Similarly to day one, the violation of the pattern resulted in a slowdown in the rate of button pressing, suggesting that younger adults were able to recall the predefined pattern. However, the RTs of older adults showed no significant change (sequential block: *M =* 0.11, SD = 0.97; random block: *M =* −0.03, SD = 0.94; *p* = 0.099) (see Supplementary Table S9). Thus unlike younger adults, older adults were not able to make use of procedural memory to recall the predefined pattern on day two.

## 4. Discussion

A child’s intelligence cannot be compressed into one stage (Piaget, 1999). Similarly, an older adult’s cognitive abilities do not diminish overnight, but deteriorate over time, with the reverse order of cognitive development (Reisberg et al., 1999). Our goal was to investigate the development and decline in declarative and procedural memory and foster an understanding of the role of retrogenesis in these processes. To our knowledge, this is the first study to examine these two fundamental learning systems across both younger and older adults in a Chinese context, let alone the retrogenic effect in age-related memory changes.

### 4.1. Age-related decline in declarative and procedural memory

Results revealed that compared to younger adults, older adults performed poorly in both declarative and procedural memory tasks, particularly in the day-two tasks. The declarative memory deterioration in older adults is likely due to the age-related reduction in the volume of the medial temporal lobe and the hippocampus, considering their vital role in declarative memory (Ullman, 2001c; Nyberg and Pudas, 2019). Converging evidence suggests age-related atrophy of the temporal lobe and hippocampus, and the shrinkage rate tended to be 2–3% per decade in the hippocampal gyrus (Cowell et al., 1994; Jack et al., 1998; Raz et al., 2004, 2005; Allen et al., 2005). In addition, older adults with declarative memory deficits were reported to exhibit disruptive alterations in neural activations and networks on both task-relevant and resting-state functional hippocampal connectivity (Pudas et al., 2013; Salami et al., 2014).

Similarly, procedural memory deterioration in older adults is likely due to atrophy in some regions of the brain, such as the basal ganglia and cerebellum, which play an important role in sequencing learning (Squire, 2004; Howard and Howard, 2013; Ullman et al., 2020). Striatal volume declines by about 3% per decade at a span between 20 and 80 years of age (Gunning-Dixon et al., 1998). There was a 2% per decade shrinkage of the vermian lobules and the cerebellar hemispheres between the ages of 20 and 80, despite spatially varying atrophy patterns within the cerebellum (Naftali et al., 2001; Han et al., 2020). However, as with younger adults, older adults seemed to be able to recruit a similar neural network of brain regions, including the cerebellum and basal ganglia, when engaging in implicit sequence learning (Daselaar et al., 2003). This may explain why older adults were able to acquire the sequential pattern in the current study, within 10 min, despite their lower learning rate.

### 4.2. Retrogenic characteristics of declarative memory decline

In the retrogenesis hypothesis for declarative memory, we hypothesized that real objects were those that had been acquired earlier and were familiar to participants, while made-up objects were first presented and later encoded into participants’ knowledge. When examining previous studies, children demonstrated better performance in recognizing real objects than in recognizing made-up objects (Hedenius et al., 2013; Lukács et al., 2017). The results of the current study revealed that older adults were also able to recognize real objects better than made-up objects in the Declearn Task. Older adults’ declarative memory performance for real objects and made-up objects was exactly the mirror pattern of that of children, see Supplementary Figure S2 for the schematic illustration.

The retrogenesis in recognizing real and made-up objects is likely due to the different patterns that declarative memory engages with these two types of objects. Real objects, for instance, are already stored in the declarative memory system, and their “consolidation process” in the brain should thus be much more robust compared to the first introduction of made-up objects (Steyvers and Tenenbaum, 2005; Kim et al., 2011). Real objects, consequently, obtain richer semantic associations, resulting in faster activation and less neural activity (Pexman et al., 2002, 2003, 2007; Kounios et al., 2009). It is reported that a significant positive correlation was observed between the age of acquisition values and gray matter density values in several regions of the brain, including the right para-hippocampal (Venneri et al., 2008), which plays an instrumental role in supporting declarative memory (Tulving and Markowitsch, 1998). According to these findings, earlier acquired objects that have been firmly ingrained in declarative memory may be more resistant to the effects of aging. However, objects which are acquired later may be stored superficially in declarative memory and thus be more susceptible to fading away from memory.

Furthermore, recognition performance across 2 days of real and made-up objects also exhibits retrogenic characteristics. Following a short delay on day one, older adults showed significantly worse results for real objects than younger adults, but marginally comparable results for made-up objects. In contrast, both real and made-up objects were recognized differently by younger and older adults after a long delay on day two. Therefore, when younger adults’ performance was used as a benchmark, older adults’ memory decayed faster for real objects versus made-up objects over 2 days. In other words, the forgetting rate of real objects is greater than that of made-up objects for older adults. The inferior performance of recognizing real objects after a short delay might be related to a hyper-binding phenomenon in which older adults encode seemingly extraneous co-occurrences in near temporal proximity and subsequently apply this knowledge to later tasks (Campbell et al., 2010). Older adults may not be able to regulate the intrusion from irrelevant distractions occurring in a close series due to their poor inhibitory control (Hasher et al., 1991; Lustig et al., 2007). Besides, there may be considerable situational and contextual connections between real objects and other entities as they were learned a long time ago (Steyvers and Tenenbaum, 2005), and these closely connected entities were likely to intrude and corrupt older adults’ recognition due to their reduced ability to down-regulate irrelevant information (Fong et al., 2021).

As another possibility, different trajectories of age-related changes in brain regions could be contributing to different types of declarative memory abilities, namely, recollection-based and familiarity-based recognition memory (Diana et al., 2007; Eichenbaum et al., 2007; Reifegerste et al., 2020). A real object involves associations with prior knowledge which are mainly retrieved through the recollection of the hippocampus (Anderson et al., 2008; Montaldi and Mayes, 2010; Barense et al., 2011; Reifegerste et al., 2020), whereas a made-up object is encoded as an isolated item and principally retrieved through the familiarity of the perirhinal cortex (Parker et al., 2002; Bowles et al., 2007; Wang et al., 2014; Reifegerste et al., 2020). In the course of aging, the hippocampal volume declines precipitously (Šimić et al., 1997; Allen et al., 2005; Raz et al., 2010; Pelletier et al., 2013; Yang et al., 2013; Koen and Yonelinas, 2014; Jack et al., 2015), but the perirhinal cortex volume declines less reliably (Insausti et al., 1998; Raz et al., 2004; Rodrigue and Raz, 2004; Daselaar et al., 2006; Koen and Yonelinas, 2014). It is consistent with the steep decline in real objects and the shallow decline in made-up objects, as also reported in previous studies in MCI patients and AD patients, for a review see Schoemaker et al. (2014) and Koen and Yonelinas (2014). And intriguingly, this general dual-process memory itself seems to follow the retrogenesis hypothesis. According to Ghetti and Lee (2013), familiarity appears to stabilize by the middle of childhood, while recollection tends to continue to improve through adolescence. When combined with our results, it is not difficult to conclude that recollection is the “last-in-first-out” ability. There is a possibility that the development and decline of the hippocampus and perirhinal cortex are at least partially responsible for this retrogenic phenomenon. The hippocampus expands until early in adolescence, but volumetric development in the perirhinal cortex terminates by age 4 (Hu et al., 2013). On the other hand, volume declines precipitously in the hippocampal region, but less reliably in the perirhinal cortex. The hippocampus is the last-developed and first-declined region, compared to the perirhinal cortex.

On the other hand, declarative memory retrogenesis should also be considered with a caveat, which is the inherent difficulty of the two types of stimuli. According to the results of this study, as well as previous studies (Hedenius et al., 2013; Lukács et al., 2017), children, older adults, and even younger adults performed better at recognizing real objects than made-up objects. This is probably because real objects that are consistent with previously acquired knowledge are more memorable than made-up objects that are inconsistent with previously acquired knowledge. Prior knowledge provides mnemonic properties that facilitate the encoding, storage, and retrieval of real objects (Badham et al., 2016). Additionally, there is a difference in difficulty based on whether or not the object can be named. Real objects can all be easily named using existing labels (Reifegerste et al., 2020). With the assistance of verbal labels, a deeper process of real objects can be promoted, resulting in more effective encoding and greater success during the later recognition phase.

### 4.3. Comparison between declarative and procedural memory declines

Although older adults exhibited a significant decline in declarative memory, they were able to recognize what they had learned 24 h previously as their performance was significantly higher than the chance level during the day-two phase. In contrast, no evidence reflected that older adults preserved sequence learning after 24 h, although younger adults did not show improvement from offline consolidation either, which is consistent with the results of Nemeth et al. (2010). In other words, with a 24-h delay, older adults could still take advantage of declarative memory but failed to make use of procedural memory. This is divergent from the expectations of the retrogenesis hypothesis that declarative memory matures later and breaks down earlier while procedural memory matures earlier and breaks down later. A possible reason for this exception is that it reflects the separability between declarative memory and procedural memory (Ullman, 2001b), and separated capacities seem to be constrained in terms of comparing their acquisition and dissolution order. Similarly, there has been some evidence of dementia patients losing their naming abilities while maintaining the ability to produce grammatical sentences (Schwartz et al., 1979), even though word meanings are acquired before the ability to produce sentences is developed. Words and sentences are processed at different levels. As Caramazza et al. (1994) pointed out, there are important “similarities” and “mutual constraints” between acquisition and breakdown, and the importance is to probe the “principles that govern the general functioning of the two systems,” rather than managing to calibrate all aspects of the two systems. The results of our study have provided direct evidence in support of the retrogenesis hypothesis in some aspects and indicated some limitations when attempting to consider acquisition and breakdown in parallel.

A limitation of the current study is that the comparison between declarative and procedural memory in older adults is not direct, although we considered the performance of younger adults as the benchmark. It would, more or less, constrain the explanatory power of the non-retrogenic results on the declarative and procedural memory. On the other hand, we only recruited one group of healthy older adults and one group of younger adults as the control to examine retrogenesis in memory decline. Future investigations of aging need to be extended to include various age groups in order to depict the continuous lifespan trajectory. Retrogenesis entails an inverted U-shaped pattern of changes (Douaud et al., 2014). An analysis of children and older adults can only probe these two ends, making it difficult to detect the nonlinear effects of aging (Veríssimo et al., 2021). Therefore, although the current study confirmed the existence of retrogenic declarative memory decline, it was insufficient to provide a systematic explanation from the perspective of retrogenesis. In addition, there is a concern about the purity of the task. For instance, as the SRT task required repetition of the sequence, participants may use subvocal rehearsal to encode the sequence (Baddeley, 1992; Baddeley, 2011). Therefore, it is likely that working memory influences the performance of the SRT task. Studies in the future should investigate the impact of working memory on the SRT task as well. Another concern is that it is not clear if older adults take advantage of compensatory mechanisms in performing the task. It is possible that the overactivation of the brain regions is responsible for the preserved performance when recognizing real objects (Reuter-Lorenz and Cappell, 2008). Future studies should record brain activity simultaneously to examine if older adults utilize additional neural circuits to maintain their “last-out” abilities.

## 5. Conclusion

To conclude, our results revealed the age-related decline in declarative and procedural memory as well as different declining trajectories of these two memory systems across short and long delays. Moreover, we provide empirical evidence to support the hypothesis that recognition failure in aging is reversed in childhood. Even though these results must be taken with caution, the patterns of development and dissolution of declarative memory in children and older adults coincide with the “last-in-first-out” trajectory, as predicted by the retrogenesis hypothesis.

## Data availability statement

The original contributions presented in the study are included in the article/Supplementary material, further inquiries can be directed to the corresponding authors.

## Ethics statement

The studies involving human participants were reviewed and approved by the Hong Kong Polytechnic University’s Human Subject Ethics Subcommittee. The patients/participants provided their written informed consent to participate in this study.

## Author contributions

CX conceived the study, developed the design, collected the data, contributed to the data analysis, the theoretical framework and interpretation of results, and the drafting of the manuscript. MF contributed to the development of the design, the data analysis, and the review of the manuscript. MM contributed to the data analysis, the interpretation of results, and the review of the manuscript. JW contributed to the review and editing of the manuscript. WW supervised the study, directed the data collection, contributed to the theoretical framework and interpretation of results, and the review of the manuscript. All authors contributed to the article and approved the submitted version.

## Funding

This work was supported by HKRGC-GRF 15601718 awarded to WW, and a Hong Kong postgraduate studentship to CX. The article processing charge was supported by the Research Institute for Smart Ageing (RISA), The Hong Kong Polytechnic University.

## Conflict of interest

The authors declare that the research was conducted in the absence of any commercial or financial relationships that could be construed as a potential conflict of interest.

## Publisher’s note

All claims expressed in this article are solely those of the authors and do not necessarily represent those of their affiliated organizations, or those of the publisher, the editors and the reviewers. Any product that may be evaluated in this article, or claim that may be made by its manufacturer, is not guaranteed or endorsed by the publisher.
