# Supplementary material for: The retrogenesis of age-related decline in declarative and procedural memory
Source: Front Psychol. 2023 Jul 27;14:1212614. doi: 10.3389/fpsyg.2023.1212614 (PMC10413564; doi:10.3389/fpsyg.2023.1212614)
Supplement: Supplementary file 1 [file Data_Sheet_1.pdf]

Supplementary Material

The retrogenesis of age-related decline in declarative and procedural memory

Chenwei Xie*, Manson Cheuk-Man Fong, Matthew King-Hang Ma, Juliahna Wang, William Shiyuan Wang

*** Correspondence:** Xie Chenwei: chenwei7.xie@connect.polyu.hk

William Shiyuan Wang: shiyuan.w.wang@polyu.edu.hk

1 Supplementary Figures and Tables

1.1 Supplementary Tables

Table SI: Group x Day ANOVA results of d-prime of declarative memory performance

Predictor	df_{Num}	df_{Den}	SS_{Num}	SS_{Den}	F	p	η^2_g
(Intercept)	1	53	156.22	20.49	404.18	.000	.83
group	1	53	12.24	20.49	31.66	.000	.28
day	1	53	4.38	10.46	22.19	.000	.12
group x day	1	53	0.05	10.46	0.24	.629	.00

Note. *dfNum* indicates degrees of freedom numerator. *dfDen* indicates degrees of freedom denominator. Epsilon indicates Greenhouse-Geisser multiplier for degrees of freedom, *p*-values and degrees of freedom in the table incorporate this correction. *SSNum* indicates sum of squares numerator. *SSDen* indicates sum of squares denominator. η2g indicates generalized eta-squared.

Table SII: Group x Condition ANOVA results of d-prime of declarative memory performance on day-one recognition phase

Predictor	df_{Num}	df_{Den}	SS_{Num}	SS_{Den}	F	p	η^2_g
(Intercept)	1	53	212.92	33.96	332.35	.000	.79
group	1	53	10.78	33.96	16.82	.000	.16
condition	1	53	49.86	21.59	122.41	.000	.47
group x condition	1	53	1.87	21.59	4.60	.037	.03

Table SIII: Post-hoc pairwise comparisons on each condition and each group of declarative memory performance on day-one recognition phase

Contrast	*estimate*	*SE*	*df*	*t.ratio*	*p*
real O – made-up O	1.086	0.174	53	6.251	<.001
real O - real Y	-0.887	0.237	53	-3.739	0.003
real O - made-up Y	0.721	0.196	53	3.675	0.003
made-up O - real Y	-1.973	0.194	53	-10.148	<.001
made-up O - made-up Y	-0.365	0.141	53	-2.584	0.059
real Y - made-up Y	1.608	0.171	53	9.425	<.001

Note. p value adjustment: tukey method for comparing a family of 4 estimates.

Table SIV: Group x Condition ANOVA results of d-prime of declarative memory performance on day-two retention phase

Predictor	df_{Num}	df_{Den}	*Epsilon*	SS_{Num}	SS_{Den}	*F*	*p*	η^2_g
(Intercept)	1.00	53.00		496.55	64.93	405.30	.000	.85
group	1.00	53.00		36.35	64.93	29.67	.000	.29
condition	1.81	95.88	0.45	43.07	25.67	88.95	.000	.32
group x condition	1.81	95.88	0.45	2.76	25.67	5.69	.006	.03

Table SV: Post-hoc pairwise comparisons on each condition and each group of declarative memory performance on day-two retention phase

Contrast	*estimate*	*SE*	*df*	*t.ratio*	*p*
real O - made-up O	0.4432	0.164	53	2.696	0.045
real O - real Y	-1.0149	0.225	53	-4.514	<.001
real O - made-up Y	0.0413	0.181	53	0.228	0.996
made-up O - real Y	-1.4581	0.179	53	-8.136	<.001
made-up O - made-up Y	-0.4018	0.12	53	-3.355	0.008
real Y - made-up Y	1.0563	0.161	53	6.542	<.001

Note. p value adjustment: tukey method for comparing a family of 4 estimates.

Table SVI: Group x Condition ANOVA results of normalized RTs of procedural memory performance on day-one phase

Predictor	df_{Num}	df_{Den}	SS_{Num}	SS_{Den}	F	p	η^2_g
(Intercept)	1	53	0.38	5.65	3.57	.064	.05
group	1	53	0.05	5.65	0.42	.518	.01
condition	1	53	2.22	1.99	59.27	.000	.23
group x condition	1	53	0.21	1.99	5.70	.021	.03

Table SVII: Post-hoc pairwise comparisons on each condition and each group of procedural memory performance on day-one phase

Contrast	*estimate*	*SE*	*df*	*t.ratio*	*p*
Random O - Sequence O	0.1961	0.0527	53	3.722	0.003
Random O - Random Y	-0.1287	0.0587	53	-2.193	0.139
Random O - Sequence Y	0.2438	0.0722	53	3.378	0.007
Sequence O - Random Y	-0.3248	0.0726	53	-4.472	<.001
Sequence O - Sequence Y	0.0477	0.0839	53	0.568	0.941
Random Y - Sequence Y	0.3725	0.0517	53	7.198	<.001

Note. *p* value adjustment: tukey method for comparing a family of 4 estimates.

Table SVIII: Group x Condition ANOVA results of normalized RTs of procedural memory performance on day-two phase

Predictor	df_{Num}	df_{Den}	SS_{Num}	SS_{Den}	F	p	η^2_g
(Intercept)	1	53	0.23	1.77	7.03	.011	.05
group	1	53	0.01	1.77	0.21	.648	.00
condition	1	53	0.30	2.42	6.68	.013	.07
group x condition	1	53	1.61	2.42	35.31	.000	.28

Table SIX: Post-hoc pairwise comparisons on each condition and each group of procedural memory performance on day-two phase

Contrast	*estimate*	*SE*	*df*	*t.ratio*	*p*
Sequence O - Random O	0.1367	0.0581	53	2.352	0.099

Sequence O - Sequence Y	0.2261	0.0567	53	3.989	0.001
Sequence O - Random Y	-0.1213	0.0537	53	-2.261	0.121
Random O - Sequence Y	0.0894	0.0535	53	1.669	0.350
Random O - Random Y	-0.258	0.0503	53	-5.127	<.001
Sequence Y - Random Y	-0.3474	0.0571	53	-6.086	<.001

Note. *p* value adjustment: tukey method for comparing a family of 4 estimates.

1.2 Supplementary Figures

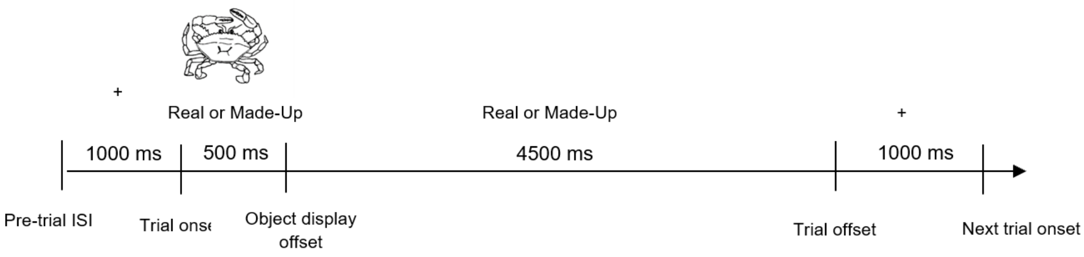

Figure SI: Sample sequence of the declearn task trials of the incidental encoding phase

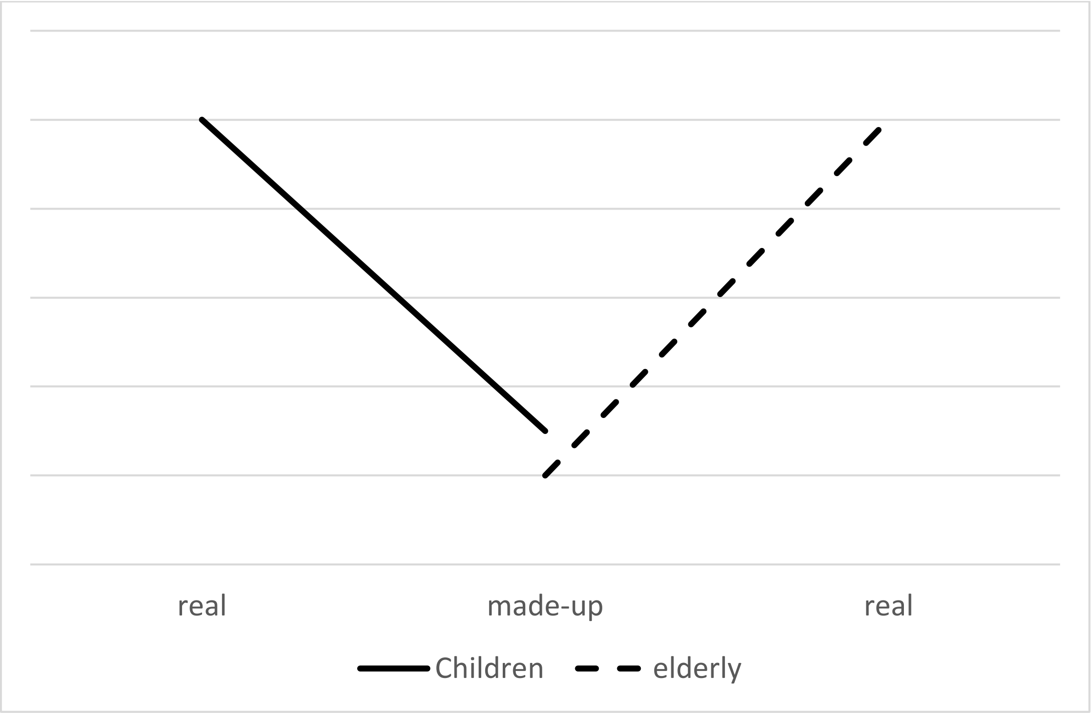

Figure SII: The schematic diagram of retrogenic declarative memory decline on real and made-up objects. The x-axis represents different types of objects. The y-axis represents normalized

performance where a higher value indicts better performance. Children acquire better DM performance in supporting real objects and it preserves better in older adults, while recognition of made-up objects scores worse and it impairs more severely in older adults. This is consistent with the retrogenesis theory that degenerative mechanisms reserve the order of acquisition in normal development.
